# Thickness of the adductor pollicis muscle in nutritional assessment of surgical patients

**DOI:** 10.1590/S1679-45082016AO3596

**Published:** 2016

**Authors:** Katarina Papera Valente, Naira Marceli Fraga Silva, Amanda Barcelos Faioli, Marina Abelha Barreto, Rafael Araújo Guedes de Moraes, Valdete Regina Guandalini

**Affiliations:** 1 Universidade Federal do Espírito Santo, Vitória, ES, Brazil.; 2 Hospital Universitário Cassiano Antonio Moraes, Vitória, ES, Brazil.

**Keywords:** Nutrition assessment, Malnutrition, Thumb/anatomy & histology, Muscles/anatomy & histology, Surgical procedures operative/methods

## Abstract

**Objective:**

To evaluate the correlation between thickness of the muscle adductor pollicis and anthropometric measurements, body mass index and Subjective Global Assessment in the nutritional assessment of surgical patients.

**Methods:**

The study population comprised patients admitted to the general and reconstructive surgery unit of a university hospital in the city of Vitória (ES), Brazil. The inclusion criteria were patients evaluated in the first 48 hours of admission, aged ≥20 years, hemodynamically stable, with no edema or ascites. Data analysis was performed using the software Statistical Package for Social Science 21.0, significance level of 5%.

**Results:**

The sample consisted of 150 patients that were candidates to surgery, mean age of 42.7±12.0 years. The most common reasons for hospitalization were surgical procedures, gastrintestinal diseases and neoplasm. Significant association was observed between thickness of adductor pollicis muscle and Subjective Global Assessment (p=0.021) and body mass index (p=0.008) for nutritional risk. Significant correlation was found between thickness of adductor pollicis muscle and arm muscle circumference, corrected arm muscle area, calf circumference and body mass index. There were no significant correlations between thickness of adductor pollicis muscle and triceps skinfold and age.

**Conclusion:**

The use of thickness of adductor pollicis muscle proved to be an efficient method to detect malnutrition in surgical patients and it should be added to the screening process of hospitalized patients, since it is easy to perform, inexpensive and noninvasive.

## INTRODUCTION

To date, there is still no accepted consensus in clinical practice as to which diagnostic tool is capable of satisfactorily identifying malnutrition in adults.^1^ In its latest update, the European Society for Clinical Nutrition and Metabolism (ESPEN) determined two alternatives for the diagnosis of malnutrition. The first takes into account only the body mass index (BMI), and the second considers the percentage of weight loss, BMI and loss of fat-free mass. This highlights the need for and the importance of body composition and anthropometric evaluations in identifying malnutrition, including patients presenting low weight or not within parameters for thinness index.^2^

The *Inquérito Brasileiro de Avaliação Nutricional Hospitalar* (IBRANUTRI) [Brazilian Inquiry for Hospital Nutritional Evaluation] assessed 4,000 inpatients at public hospitals in large Brazilian cities, and showed 1,924 patients (48.1%) presented with some degree of malnutrition. Among these malnourished patients, 504 (12.6%) were severely malnourished, and 1,420 (35.5%) were moderately malnourished.^3^ In Brazil, an average of 15 to 20% of inpatients are malnourished upon admission, due to several factors, such as underlying disease, poor social and economic conditions, and healthcare system deficiencies.^4^

There are various methods to evaluate inpatients, including dietary history, anthropometric and biochemical data, clinical history and physical examination, with specific limitations, advantages, and disadvantages. Anthropometric data are frequently used for nutritional assessment of inpatients, although there is no gold standard that is completely accurate.^4^ Among the conventional anthropometric measurements, assessment of the adductor pollicis muscle thickness (APMT) is an important variable to assess the muscle compartment, since it is considered an objective, quick, and low-cost measurement, besides being noninvasive.^5^

The measurement of the adductor pollicis muscle of the dominant hand is always superior relative to the adductor pollicis muscle of the non-dominant hand, due to the fact of its being the first to suffer the influence of daily activities, which are preferably performed by this limb. Therefore, it is better to measure the non-dominant hand, for the most activated muscles tend to present atrophy more rapidly in a situation of malnutrition, and may not faithfully represent the nutritional status.^6^

Melo et al.,^7^ carried out a study with 151 patients who are candidates for elective surgical procedures in order to estimate the prevalence of malnutrition using the APMT. The classic anthropometric measurements were used, along with percentage of weight loss and measurement of the adductor pollicis muscle in both hands. In this study, a high prevalence of malnutrition was found, besides a significant association between the nutritional diagnosis as per the APMT and the measurement of the arm circumference (AC), triceps skinfold (TS), and BMI, showing that the adductor muscle appears as a good method for diagnosing muscle depletion and malnutrition in surgical patients.

Associated with the anthropometric measurements, screening and subjective nutritional evaluation are used to detect patients at risk of malnutrition upon admission. Among them, especially the Subjective Global Assessment (SGA) and the Nutritional Risk Screening stand out. These protocols should be applied and used in the first hours of intervention, seeking an early detection of malnutrition, so that intervention might begin quickly, improving the patient’s general status and thus decreasing expenses related to hospital stay. SGA classifies the patients as well- nourished, moderately malnourished, and severely malnourished; on the other hand, the Nutritional Risk Screening classifies the priority of nutritional intervention.^8^ For this study, we chose to work with SGA, since it is considered the gold-standard procedure in nutritional assessment of inpatients.^9^ However, adequate training of all observers who desire to use it is crucial, because its precision depends on the capacity of the observer to detect significant nutritional alterations by means of subjective evaluation.^10^

Faced with this and the many nutritional evaluation methods that already exist, further studies are needed, with simple and practically noninvasive methods, such as the APMT, in order to implement new tools that might accelerate the identification of nutritional risk.

## OBJECTIVE

To evaluate the correlation between thicknesses of adductor pollicis muscle with anthropometric measurements, body mass index, and Subjective Global Assessment in the nutritional diagnosis of surgical patients.

## METHODS

This is a cross-sectional study carried out between August 2014 and March 2015, at a General and Reconstructive Surgery Unit of a university hospital located in the city of Vitória (ES). This study is a part of the research project entitled “Malnutrition and associated factors at a university hospital of the Greater Vitória Area (ES)”, approved by the Research Ethics Committee of the *Universidade Federal do Espírito Santo*, under number CAAE: 27954014.0.0000.5060.

The participants of the study were all patients admitted to the two units abovementioned, who could be evaluated within the first 24 and 48 hours after admission and who met the following inclusion criteria: aged ≥20 years; hemodynamically stable; no edema and ascites; and agreed to participate by signing the Informed Consent Form (ICF).

For nutritional status evaluation, the following parameters were used: conventional anthropometric data, APMT, and SGA.

### Anthropometric evaluation

The patients included in the study answered a structured questionnaire to gather social and demographic data, followed by SGA and the collection of anthropometric data by previously trained evaluators, based on a pilot study. The clinical history was collected from the medical records in patient´s charts.

For the nutritional status evaluation, current weight, height, calf circumference (CC), AC, TS, arm muscle circumference (AMC), corrected arm muscle area (CAMA), and BMI.

For the anthropometric evaluation, data were collected regarding weight (in kilograms) and height (in meters), according to the techniques proposed by Lohman et al.^11^ Weight was checked using a tetrapolar Tanita^®^ bioimpedence scales, with maximum capacity for 150kg and precision of 100g. For the measurement of height, the AlturExata^®^ portable stadiometer, with bilateral scales in millimeters and capacity for use of 0.35 to 2.13m was employed. For bed-ridden patients, reclined height and estimated body weight were considered, by means of the equation by Chumlea et al.,^12^ for both genders and cycle of life.

AMC was obtained based on the values of AC and TS, by means of equation 1:


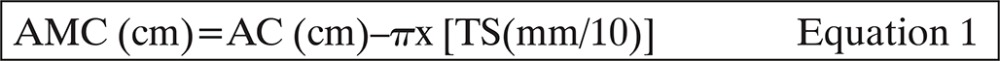


Corrected arm muscle area was calculated based on equation 2, corrected for each gender.


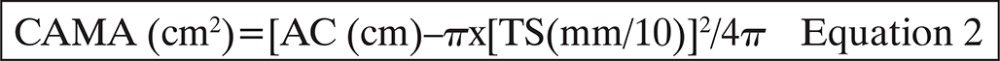


For the classification of AMC and CAMA, the percentage values proposed by Frisancho were used.^13^

Calf circumference was determined using an inelastic tape measure horizontally around the maximum circumference.^14^ Values below 31cm were considered indicative of reduced muscle mass.^15^

Body mass index was calculated based on the formula current weight (kg)/height^2^ (m). The adults were classified as per the references of the World Health Organization,^16^ considering the following intervals: low weight, if BMI<18.5kg/m^2^; eutrophic, if BMI≥18.5kg/m^2^ to 24.9kg/m^2^; overweight, if BMI>24.9kg/m^2^ to 29.9kg/m^2^; and obesity if BMI>29.9kg/m^2^. The elderly were classified according to the cutoff points of Lipschitz,^17^ in which low weight was BMI≤22kg/m^2^; eutrophic if BMI was between 22kg/m^2^ and 27kg/m^2^; and overweight, if BMI>27kg/m^2^.

### Adductor pollicis muscle thickness

Adductor pollicis muscle thickness was measured while the patient sitting down, the arm flexed at approximately 90°, the forearm and the hand supported on the knee. The patients were instructed to keep their hands relaxed. A Cescorf^®^ (Porto Alegre, RS, Brazil), skinfold caliper was used with continuous pressure of 10g/mm^2^ to pinch the adductor muscle at the vertex of an imaginary triangle formed by the extension of the thumb and the index finger.^18^ The procedure was done on the non-dominant hand three times, and the mean was used as measurement of the APMT. To classify the values obtained, the proposal of Bragagnolo et al.,^5^ specific for surgical patients, was used; it considers values of eutrophy for APMT of the non-dominant hand >13.1mm and of malnutrition, values <13.1mm.

### Subjective Global Assessment

The SGA was initially proposed to assess the nutritional status of inpatients during the postoperative period, and has been used in several different clinical settings, considered as gold standard for this type of evaluation. It may be considered a marker of health status, in which the diagnosis of severe malnutrition is an indicator of severity of disease and not only an index of nutrient *deficit*.^9^

The SGA focuses on aspects of the clinical history, such as weight changes, intake alterations, presence of gastrintestinal symptoms, and alterations in functional capacity, besides evaluating, in the physical examination, subcutaneous fat and muscle mass losses, presence of sacral edema, ankle edema, and ascites. The results are expressed in three categories: well-nourished patients (SGA “A”), with suspect/moderate malnutrition (SGA “B”), or severe malnutrition (SGA “C”).

### Statistical analysis

The data obtained were stored in an electronic Microsoft Excel 2007^®^ spreadsheet, and posteriorly, the statistical analysis was performed using the Statistical Package for Social Science software, version 21.0. To describe the study variables, measurements of central tendency (mean) and of dispersion (standard deviation) for the continuous variables, and percentages for the categorical variables were used. Normality of the distribution of the variables was assessed by the Kolmogorov-Smirnov test. All variables presented normal distribution. For data analysis, SGA was classified based on the nutritional risk in well-nourished patients (SGA A) and in malnourished patients (SGA B and C). Pearson’s χ^2^ test was used to analyze the differences between the categorical variables, as well as the correlation among the continuous variables. The multiple linear regression analysis (stepwise method) was applied to determine which independent variables were associated with the APMT (dependent variable).

## RESULTS

Of 150 patients evaluated, 110 (73.3%) were adults and 40 (26.7%) elderly, with mean ages of 42.7±12.0 years. As to gender, 84 (56.0%) were male. There was a predominance of the mulatto ethnic group, represented by 67 (44.6%) individuals. As to the clinical diagnosis and surgical indication, we draw attention to neoplasms, which affected 37 patients (24.6%), followed by 36 (24.0%) patients with gastrintestinal and pancreatic diseases, and 35 (23.3%) patients with hepatobiliary diseases. Other variables that characterize the sample are presented on table 1.

**Table 1:** Characterization of the sample according to life stage, gender, ethnicity, and clinical diagnosis

Variable	n (%)
Stage of life	
Adult	110 (73.3)
Elderly	40 (26.7)
Gender	
Male	84 (56.0)
Female	66 (44.0)
Ethnicity	
Mulatto	67 (44.6)
White	60 (40.0)
Black	17 (11.4)
Yellow	6 (4.0)
Diagnosis	
Neoplasims	37 (24.6)
GI/pancreatic diseases	36 (24.0)
Hepatobiliary diseases	35 (23.3)
Inguinal hernia	20 (13.3)
Cardiovascular diseases	11 (7.3)
Pulmonary diseases	6 (4.0)
Others	5 (3.3)

GI: gastrintestinal.

A predominance of excess weight (74.4%) was noted by the BMI, and of well-nourished by the SGA (72.0%). When considering the APMT, there was also a predominance of well-nourished (60.0%) (Table 2).

**Table 2:** Nutritional status of the sample according to body mass index, Subjective Global Assessment, and adductor pollicis muscle thickness

	n (%)
APMT	
Malnourished	60 (40.0)
Well-nourished	90 (60.0)
BMI	
Low weight	24 (16.0)
Eutrophic	55 (36.6)
Excess weight	71 (74.4)
SGA	
Well-nourished	108 (72.0)
Moderately malnourished	11 (7.3)
Severely malnourished	31 (20.7)

APMT: adductor pollicis muscle thickness; BMI: body mass index; SGA: Subjective Global Assessment.

Table 3 presents the distribution of nutritional status according to the adductor pollicis muscle thickness of the non-dominant hand, relative to gender, stage of life, BMI, SGA, and diagnosis. As to APMT, eutrophy proved more frequent in men and malnutrition in women, with a significant difference (p=0.004). A significnat association was found between the nutritional status defined by the APMT with the SGA (p=0.021) and with the BMI, with nutritional risk (p=0.007). Stage of life, BMI with no nutritional risk, and diagnosis showed no association with the nutritional status indicated by the measurement of the APMT.

**Table 3:** Frequency of nutritional status as per adductor pollicis muscle thickness of the non-dominant hand, according to gender, stage of life, body mass index, and diagnosis

	APMT		Total n (%)	p value

	Malnourished (<13.1 mm) n (%)	Eutrophic (>13.1 mm) n (%)		
Gender				
Male	23 (27.4)	61 (72.6)	84 (100)	0.000*
Female	37 (56.0)	29 (44.0)	66 (100)	
Stage of life				
Adult	46 (41.8)	64 (58.2)	110 (100)	0.523
Elderly	14 (35.0)	26 (65.0)	40 (100)	
BMI				
With risk	17 (65.4)	9 (34.6)	26 (100)	0.004*
Without risk	43 (34.7)	81 (65.3)	124 (100)	
SGA				
With risk	37 (34.3)	71 (65.7)	108 (100)	0.026*
Without risk	23 (54.8)	19 (45.2)	42 (100)	
Diagnosis				
Neoplasm	16 (43.2)	21 (56.8)	37 (100)	0.288
Gastrintestinal diseases	15 (41.7)	21 (58.3)	36 (100)	
Hepatobiliary diseases	12 (34.3)	23 (65.7)	35 (100)	
Inguinal hernia	6 (30.0)	14 (70.0)	20 (100)	
Cardiovascular diseases	8 (72.7)	3 (27.3)	11 (100)	
Pulmonary diseases	2 (33.3)	4 (66.7)	6 (100)	
Other	1 (20.0)	4 (80.0)	5 (100)	

*Pearson’s χ^2^; p<0.05*. APMT: adductor pollicis muscle thickness; BMI: body mass index; SGA: Subjective Global Assessment.

The correlations between APMT and age, TS, AMC, (CAMA), CC, and BMI were analyzed. Significant correlations were found, although weak, between APMT and the anthropometric, AMC, (CAMA), CC, and BMI variables. No significant correlations were found between APMT and TS and age (Table 4).

**Table 4:** Correlation between the thickness of the adductor pollicis muscle and classic nutritional status variables

Variables (n=150)	Mean	SD	95% CI		r^*^

			Lower	Upper	
Age	49.93	16.05	47.34	52.52	0.122
TS	16.46	8.35	15.12	17.81	0.105
AMC	23.57	3.35	23.03	24.11	0.326†
CAMA	36.66	12.43	34.65	38.67	0.371†
CC	34.47	4.62	33.72	35.22	0.320†
BMI	25.17	5.09	24.34	25.99	0.290†

* Pearson’s correlation test; † p<0.01. SD: standard deviation; 95% CI: 95% confidence interval; TS: triciptal skinfold; AMC: arm muscle circumference; CAMA: corrected arm muscle area; CC: calf circunference; BMI: body mass index.

Table 5 displays the multiple linear regression results. In the final model, the variables gender, AMC, CAMA, and BMI remained, explaining 24% of APMT. AMC was the variable that most influenced our measurement, even after the adjustment for gender, CAMA, and BMI, with a reduction of 0.392mm.

**Table 5:** Multiple linear regression for the dependent variable of adductor pollicis muscle thickness

	APMT		

	β	Standard error	p value
Gender	0.300	0.795	0.001
AMC	-0.392	0.249	0.036
CAMA	0.491	0.061	0.004
BMI	0.305	0.092	0.004

R^2^=0.238. APMT: adductor pollicis muscle thickness; AMC: arm muscle circumference; CAMA: corrected arm muscle area; BMI: body mass index.

## DISCUSSION

In this study, the nutritional status defined based on the BMI and the SGA showed a significant correlation with the APMT. After adjustment for the confounding variables, the APMT was associated with gender, AMC, CAMA, and BMI. There were significant differences between males and females, but not among adults and the elderly.

Melo et al.,^7^ found malnutrition in 31.8% of male patients and in 68.2% of females. These data corroborate with our study and other similar studies^18-20^ in which men presented greater anthropometric measurements.

Adductor pollicis muscle thickness is capable of estimating muscle mass loss and it has some advantages, such as it is easy, quick, and cheap to measure, besides eliminating the use of formulas to calculate the muscle compartment.^19^ Some studies^5,21-23^ demonstrated its use in many clinical conditions and treatments, with significant results regarding nutritional diagnosis, correlation with anthropometric variables, lean mass reduction, and association in prognosis of complications in the postoperative period and during hospital stay.^6,24^

The results found here present evidence that the APMT can be used as an indicator of nutritional status and of muscle mass reduction, since it remained associated with other anthropometric measurements that evaluated the same compartment. That is the case of AMC and CAMA, in addition to ratifying the importance of its use in the preoperative period to identify the nutritional risk and early nutritional intervention.

The APMT can indicate changes in body composition, and therefore, be useful in detecting early alterations related to malnutrition and evaluate the nutritional recovery.^20^

A study carried out by Bragagnolo et al.^25^ assessed 124 patients submitted to major gastrintestinal tract surgeries and concluded that the APMT can be used as a predictor of postoperative complications and mortality, besides being an important tool to assess the nutritional status of surgical patients. This study further evaluated the association between APMT and SGA, which is considered a gold standard method in the nutritional assessment of hospitalized patients. The results showed a significant correlation between them, although the SGA did not remain in the final model by regression analysis. AMC was the variable that most influenced in the measurement of the APMT in this population, after adjustments.

A study by da Silva et al.^26^ assessed the nutritional status of 43 oncology patients, aged over 18 years, considering APMT, BMI, TS, AC, CAMA, and the palmar prehension strength of the patients. There was little agreement among the parameters used, such as APMT and SGA, but significant differences were found among the values of AC, CAMA, APMT, and palmar prehension strength, indicating that these parameters may be useful in the identification of well-nourished and mal-nourished patients, as long as the cutoff points are defined.

Different results were found by Gonzalez et al.,^27^ who analyzed 361 surgical patients. After the analysis for confounding variables, the nutritional status evaluated by the SGA was a determinant factor in the measurement da APMT, with a significant reduction of its values in moderately and severely malnourished patients. Similar finding were identified by Bragagnolo et al.^5^ and Caporossi et al.^22^

Poziomyck et al.^28^ studied 74 adult and elderly patients submitted to resection of gastrintestinal tumors, with the objective of evaluating which would be the most sensitive nutritional assessment method in this group. They used SGA, APMT, BMI, AC, AMC, percentage of weight loss, and TS, in addition to biochemical tests. The results revealed that the APMT and SGA are reliable in predicting mortality, and can be used in clinical practice.

There is consensus in the literature as to the ease of application, low cost, location of the muscle evaluated, and the several applications in which APMT is possible. However, it should be pointed out that the absence of a reference standard for the various clinical conditions, gender, and life cycles can generate results that are not representative of the true nutritional status, clinical condition and hydration status of the patient.

Atrophy of the APMT reflects loss of productive life. The presence of malnutrition and the underlying disease may cause reduced daily activities and possible catabolism, resulting in progressive decrease of the APMT.^22^

According to Gonzalez et al.,^20^ it is important to point out that investigations that identify discrepant values relative to the references indicated in literature may be based on error, at the time of pinching the correct anatomic landmark, in calibration of the skinfold caliper, as well as in the variability among raters of the same study.

Among the limitations of this study, it should be highlighted the fact of being a cross-sectional study carried out in a single unit, with high turnover and specific characteristics. Moreover, it did not consider the biochemical alterations and it was an observer-dependent research.

## CONCLUSION

The results found indicated that measurement of adductor pollicis muscle thickness can be included in the nutritional triage of surgical patients, aiming to speed up and facilitate the nutritional diagnosis of these patients, and to detect protein depletion. The adductor pollicis muscle thickness proved to be a reliable measurement, which can be implemented in the process of nutritional assessment, for being capable of identifying the risk of malnutrition, along with other variables and nutritional evaluation methods. Nevertheless, further investigations are required to identify the reasons for different findings in the literature, especially as to the cutoff point to assess surgical patients, taking into account the age range and gender.
