# Supplementary material for: Thickness of the adductor pollicis muscle in nutritional assessment of surgical patients
Source: Einstein (Sao Paulo). 2016 Jan-Mar;14(1):18–24. doi: 10.1590/S1679-45082016AO3596 (PMC4872912; doi:10.1590/S1679-45082016AO3596)
Supplement: Translation

# Translation: Espessura do músculo adutor do polegar na avaliação nutricional de pacientes cirúrgicos

**Authors:** Katarina Papera Valente, Naira Marceli Fraga Silva, Amanda Barcelos Faioli, Marina Abelha Barreto, Rafael Araújo Guedes de Moraes, Valdete Regina Guandalini

**Affiliations:** 1 Universidade Federal do Espírito Santo, Vitória, ES, Brasil.; 2 Hospital Universitário Cassiano Antonio Moraes, Vitória, ES, Brasil.

**Keywords:** Avaliação nutricional, Desnutrição, Polegar/anatomia & histologia, Músculos/anatomia & histologia, Procedimentos cirúrgicos operatórios/métodos

## Abstract

**Objetivo:**

Avaliar a correlação entre a medida da espessura do músculo adutor do polegar com medidas antropométricas, índice de massa corporal e Avaliação Subjetiva Global no diagnóstico nutricional de pacientes cirúrgicos.

**Métodos:**

Participaram do estudo pacientes internados em uma unidade de cirurgia geral e reparadora de um hospital universitário de Vitória (ES), Brasil. Os critérios de inclusão foram pacientes avaliados nas primeiras 48 horas de internação, idade ≥20 anos hemodinamicamente estáveis, sem edema ou ascite. A análise dos dados foi realizada pelo *softwareStatistical Package for Social Science*, versão 21.0, com nível de significância de 5%.

**Resultados:**

Compuseram a amostra 150 pacientes candidatos à cirurgia, com média de idade de 42,7±12,0 anos. Os motivos de internação mais comuns foram procedimentos cirúrgicos, doenças do trato gastrointestinal e neoplasias. Observou-se associação significativa entre a espessura do músculo adutor do polegar com a Avaliação Subjetiva Global (p=0,021) e com o índice de massa corporal para risco nutricional (p=0,008). Foram encontradas correlações significativas entre a espessura do músculo adutor do polegar com circunferência muscular do braço, área muscular do braço corrigida, circunferência da panturrilha e índice de massa corporal. Não foram encontradas correlações significativas entre a espessura do músculo adutor do polegar com prega cutânea triciptal e idade.

**Conclusão:**

O uso da espessura do músculo adutor do polegar mostrou-se um método eficiente na detecção de desnutrição em pacientes cirúrgicos e deve ser acrescentado ao processo de triagem de pacientes hospitalizados, uma vez que é de fácil execução, baixo custo e não invasivo.

## INTRODUÇÃO

Ainda não há um consenso aceito na prática clínica sobre qual ferramenta diagnóstica é capaz de identificar, de maneira satisfatória, a desnutrição no adulto.^1^ A *European Society for Clinical Nutrition and Metabolism* (ESPEN), em sua última atualização, determinou duas alternativas para o diagnóstico de desnutrição. A primeira leva em consideração somente o índice de massa corporal (IMC), e a segunda considera o porcentual de perda de peso, o IMC e a perda de massa livre de gordura. Dessa forma, ressaltam-se a necessidade e a importância da avaliação antropométrica e da composição corporal para identificar a desnutrição, mesmo em pacientes fora do índice de magreza ou baixo peso.^2^

O Inquérito Brasileiro de Avaliação Nutricional Hospitalar (IBRANUTRI), avaliou 4.000 pacientes internados em hospitais da rede pública de grandes cidades brasileiras, e revelou que 1.924 pacientes (48,1%) apresentaram algum grau de desnutrição. Entre esses pacientes desnutridos, 504 (12,6%) eram desnutridos graves e 1.420 (35,5%) eram desnutridos moderados.^3^ No Brasil, em média, 15 a 20% dos pacientes são internados desnutridos, devido a vários fatores, como doença de base, más condições socioeconômicas e a deficiência do sistema de saúde.^4^

Existem diversos métodos para avaliação do paciente hospitalizado, como anamnese alimentar, dados antropométricos e bioquímicos, história clínica e exame físico, com limitações, vantagens e desvantagens específicas. A antropometria é frequentemente utilizada para avaliação nutricional desse grupo, embora não haja um padrão-ouro e totalmente preciso.^4^ Entre as medidas antropométricas convencionais, a avaliação da espessura do músculo adutor do polegar (EMAP) aparece como uma variável importante para avaliar o compartimento muscular, pois é considerada uma medida objetiva, rápida e de baixo custo, além de não invasiva.^5^

A medida do músculo adutor do polegar da mão dominante mostra-se sempre superior em relação à do músculo adutor do polegar da mão não dominante, devido ao fato de o primeiro sofrer influência das atividades diárias, preferencialmente desenvolvidas nesse membro. Assim, prefere-se medir a mão não dominante, uma vez que a musculatura mais exercitada tende a atrofiar mais rapidamente em situação de desnutrição, podendo não representar fielmente a condição nutricional.^6^

Melo et al.,^7^ realizaram um estudo com 151 pacientes candidatos a procedimentos cirúrgicos eletivos para estimar a prevalência de desnutrição pela EMAP. Foram utilizadas medidas antropométricas clássicas, porcentual de perda de peso e medida do músculo adutor do polegar em ambas as mãos. Nesse estudo, foi encontrada alta prevalência de desnutrição, além de associação significativa entre o diagnóstico nutricional segundo EMAP e as medidas de circunferência do braço (CB), prega cutânea tricipital (PCT) e o IMC, mostrando que o músculo adutor aparece como um bom método para diagnosticar depleção muscular e desnutrição em pacientes cirúrgicos.

Associados às medidas antropométricas, os métodos de triagem e avaliação subjetiva nutricional são utilizados para detectar pacientes em risco de desnutrição no momento da internação. Entre eles, destacam-se a Avaliação Subjetiva Global (ASG) e a *Nutritional Risk Screening*. Esses protocolos devem ser aplicados e utilizados nas primeiras horas de internação, visando a uma detecção precoce de desnutrição, para que a intervenção se inicie rapidamente, melhorando o estado de saúde geral do paciente e diminuindo, assim, gastos relacionados à internação hospitalar. ASG classifica os pacientes como bem-nutridos, moderadamente desnutridos e gravemente desnutridos; já a *Nutritional Risk Screening* classifica a prioridade de intervenção nutricional_._^8^ Para este estudo, optou-se por trabalhar com a ASG, por ser um procedimento considerado padrão-ouro na avaliação nutricional de pacientes hospitalizados,^9^ mas faz-se indispensável o treinamento adequado de todos os observadores que desejam praticá-la, pois sua precisão depende da capacidade do observador detectar as alterações nutricionais significativas por meio da avaliação subjetiva.^10^

Diante disso e dos muitos métodos de avaliação nutricional já existentes, são necessários estudos com métodos simples e pouco invasivos, como é a medida da EMAP, para implementar novas ferramentas que possam acelerar a identificação do risco nutricional.

## OBJETIVO

Avaliar a correlação entre a medida da espessura do músculo adutor do polegar com medidas antropométricas, índice de massa corporal e Avaliação Subjetiva Global no diagnóstico nutricional de pacientes cirúrgicos.

## MÉTODOS

Trata-se de um estudo transversal, por conveniência, realizado entre agosto de 2014 e março de 2015, em uma Unidade de Cirurgia Geral e Reparadora de um hospital universitário localizado em Vitória (ES). Este estudo faz parte do projeto de pesquisa intitulado “Desnutrição e fatores associados em um hospital universitário da Grande Vitória, (ES)”, aprovado pelo Comitê de Ética e Pesquisa da Universidade Federal do Espírito Santo, sob o número do CAAE: 27954014.0.0000.5060.

Participaram do estudo todos os pacientes admitidos nas duas enfermarias da unidade citada, que pudessem ser avaliados nas primeiras 24 e 48 horas de admissão hospitalar e que atendessem aos seguintes critérios de inclusão: idade ≥20 anos de idade; estar hemodinamicamente estável; não apresentar edemas e ascite; e consentir sua participação por meio da assinatura do Termo de Consentimento Livre e Esclarecido (TCLE).

Para a avaliação do estado nutricional, foram utilizados os seguintes parâmetros: antropometria convencional, EMAP e ASG.

### Avaliação antropométrica

Os pacientes incluídos no estudo responderam a um questionário estruturado para coleta de dados sociodemográficos, seguido da ASG e da coleta de dados antropométricos por avaliadores previamente treinados, a partir de um estudo piloto. A história clínica foi coletada nos registros médicos contidos em prontuários.

Para a avaliação do estado nutricional, foram utilizados peso atual, estatura, circunferência da panturrilha (CP), CB, PCT, circunferência muscular do braço (CMB), área muscular do braço corrigida (AMBc) e IMC.

Para a avaliação antropométrica, foram coletados dados de peso (em quilograma) e estatura (em metro), de acordo com as técnicas propostas por Lohman et al.^11^ O peso foi aferido com o auxílio de uma balança de bioimpedância tetrapolar Tanita^®^, com capacidade máxima de 150kg e precisão de 100g. Para a medida da estatura, foi utilizado o estadiômetro portátil AlturExata^®^, com escala bilateral em milímetros e capacidade de uso de 0,35 a 2,13m. Para os pacientes acamados, foi considerada a estatura recumbente e a estimativa de peso corporal, por meio da equação de Chumlea et al.,^12^ para ambos os gêneros e ciclo da vida.

A CMB foi obtida a partir dos valores de CB e PCT, por meio da equação 1:



A AMBc foi calculada a partir da equação 2, corrigida para cada gênero.



Para a classificação da CMB e da AMBc, foram utilizados os valores em percentil propostos por Frisancho.^13^

A CP foi determinada utilizando-se fita inelástica horizontalmente em volta da circunferência máxima.^14^ Valores abaixo de 31cm foram considerados indicativos de redução de massa muscular.^15^

O IMC foi calculado a partir da fórmula peso atual (kg)/estatura^2^ (m). Os adultos foram classificados de acordo com as referências da Organização Mundial da Saúde,^16^ considerando os seguintes intervalos: baixo peso, se IMC<18,5kg/m^2^; eutrofia, se IMC≥18,5 a 24,9kg/m^2^; sobrepeso, se IMC>24,9kg/m^2^ a 29,9kg/m^2^; e obesidade, se IMC>29,9kg/m^2^. Os idosos foram classificados segundo os pontos de corte de Lipschitz,^17^ sendo baixo peso se IMC≤22kg/m^2^; eutrofia, se IMC entre 22kg/m^2^ e 27kg/m^2^; e sobrepeso, se IMC>27kg/m^2^.

### Espessura do músculo adutor do polegar

A medida da EMAP foi realizada com o paciente sentado, o braço flexionado a aproximadamente 90°, o antebraço e a mão apoiados sobre o joelho. Os pacientes foram orientados a ficar com a mão relaxada. Foi utilizado o plicômetro da marca Cescorf^®^ (Porto Alegre, RS, Brasil), com pressão contínua de 10g/mm^2^ para pinçar o músculo adutor no vértice de um triângulo imaginário, formado pela extensão do polegar e indicador.^18^ O procedimento foi feito na mão não dominante por três vezes, sendo usada a média como medida da EMAP. Para classificação dos valores obtidos, foi utilizada a proposta de Bragagnolo et al.,^5^ específica para pacientes cirúrgicos, que considera valores de eutrofia para EMAP da mão não dominante >13,1mm e, de desnutrição, valores <13,1mm.

### Avaliação Subjetiva Global

A ASG foi inicialmente proposta para avaliação do estado nutricional de pacientes hospitalizados no pós-operatório e vem sendo utilizada em diversas condições clínicas, considerada como padrão-ouro para esse tipo de avaliação. Pode ser considerado um marcador do estado de saúde, sendo o diagnóstico da desnutrição grave um indicador de gravidade da doença, e não apenas um índice do *déficit* de nutrientes.^9^

A ASG contempla aspectos da história clínica, como mudanças de peso, alterações da ingestão alimentar, presença de sintomas gastrointestinais e alterações da capacidade funcional, além de avaliar, no exame físico, a perda de gordura subcutânea e de massa muscular, a presença de edema sacral, de edema de tornozelo e ascite. Os resultados são expressos em três categorias: pacientes bem-nutridos (ASG “A”), com desnutrição suspeita/moderada (ASG “B”) ou com desnutrição grave (ASG “C”).

### Análise estatística

Os dados obtidos foram armazenados numa planilha eletrônica no *software* Microsoft Excel 2007^®^ e, posteriormente, foi realizada a análise estatística no *softwareStatistical Package for Social Science*, versão 21.0. Para descrever as variáveis de estudo, foram utilizadas medidas de tendência central (média) e de dispersão (desvios padrão) para as variáveis contínuas, e porcentuais para as variáveis categóricas. A normalidade da distribuição das variáveis foi avaliada pelo teste de Kolmogorov-Smirnov. Todas as variáveis apresentaram distribuição normal. Para análise dos dados, a ASG foi categorizada com base no risco nutricional em pacientes bem nutridos (ASG A) e pacientes desnutridos (ASG B e C). Para análise das diferenças entre as variáveis categóricas, foi aplicado o teste χ^2^ de Pearson. Para verificar a correlação entre as variáveis contínuas, foi utilizada a correlação de Pearson. A análise de regressão linear múltipla (método *stepwise*) foi aplicada para determinar quais variáveis independentes associaram-se ao EMAP (variável dependente).

## RESULTADOS

Dos 150 pacientes avaliados, 110 (73,3%) eram adultos e 40 (26,7%) idosos, com média de idade de 42,7±12,0 anos. Quanto ao gênero, 84 (56,0%) eram do gênero masculino. Houve predomínio de etnia parda, representada por 67 (44,6%) dos indíviduos. Quanto ao diagnóstico clínico e à indicação cirúrgica, destacaram-se as neoplasias, que acomenteram 37 pacientes (24,6%), seguidas por 36 (24,0%) pacientes com doenças do trato gastrointestinal e pancreáticas, e 35 (23,3%) pacientes com doenças hepatobiliares. Demais variáveis de caracterização da amostra estão apresentadas na tabela 1.

**Tabela 1:** Caracterização da amostra segundo o estágio de vida, gênero, etnia e diagnóstico clínico

Variável	n (%)
Estágio da vida	
Adulto	110 (73,3)
Idoso	40 (26,7)
Gênero	
Masculino	84 (56,0)
Feminino	66 (44,0)
Etnia	
Pardo	67 (44,6)
Branco	60 (40,0)
Preto	17 (11,4)
Amarelo	6 (4,0)
Diagnóstico	
Neoplasias	37 (24,6)
Doenças do TGI/pancreáticas	36 (24,0)
Doenças hepatobiliares	35 (23,3)
Hérnia inguinal	20 (13,3)
Doenças cardiovasculares	11 (7,3)
Doenças pulmonares	6 (4,0)
Outras	5 (3,3)

TGI: trato gastrointestinal.

Foi identificado predomínio de excesso de peso (74,4%) pelo IMC e de bem nutridos pela ASG (72,0%). Quando avaliada a medida da EMAP, também houve predomínio da classificação bem nutrido (60,0%) (Tabela 2).

**Tabela 2:** Estado nutricional da amostra segundo índice de massa corporal, Avaliação Subjetiva Global e espessura do músculo adutor do polegar

	n (%)
EMAP	
Desnutrido	60 (40,0)
Bem nutrido	90 (60,0)
IMC	
Baixo peso	24 (16,0)
Eutrofia	55 (36,6)
Excesso de peso	71 (74,4)
ASG	
Bem nutrido	108 (72,0)
Moderadamente desnutrido	11 (7,3)
Gravemente desnutrido	31 (20,7)

EMAP: espessura do músculo adutor do polegar; IMC: índice de massa corporal; ASG: Avaliação Subjetiva Global.

A tabela 3 apresenta a distribuição do estado nutricional segundo a espessura do músculo adutor do polegar da mão não dominante, em relação a gênero, estágio da vida, IMC, ASG e diagnóstico. Quanto à EMAP, a eutrofia mostrou-se mais frequente em homens e a desnutrição, em mulheres, com diferença significativa (p=0,004). Foi observada associação significativa entre o estado nutricional definido pela EMAP com a ASG (p=0,021) e com o IMC, com risco nutricional (p=0,007). Estágio da vida, IMC sem risco nutricional e diagnóstico não mostraram associação com o estado nutricional indicado pela medida da EMAP.

**Tabela 3:** Frequência do estado nutricional pela espessura do músculo adutor do polegar da mão não dominante segundo gênero, estágio de vida, índice de massa corporal e diagnóstico

	EMAP		Total n (%)	Valor de p

	Desnutrido (<13,1mm) n (%)	Eutrófico (>13,1mm) n (%)		
Gênero				
Masculino	23 (27,4)	61 (72,6)	84 (100)	0,000*
Feminino	37 (56,0)	29 (44,0)	66 (100)	
Estágio da vida				
Adulto	46 (41,8)	64 (58,2)	110 (100)	0,523
Idoso	14 (35,0)	26 (65,0)	40 (100)	
IMC				
Com risco	17 (65,4)	9 (34,6)	26 (100)	0,004*
Sem risco	43 (34,7)	81 (65,3)	124 (100)	
ASG				
Com risco	37 (34,3)	71 (65,7)	108 (100)	0,026*
Sem risco	23 (54,8)	19 (45,2)	42 (100)	
Diagnóstico				
Neoplasias	16 (43,2)	21 (56,8)	37 (100)	0,288
Doenças do trato gastrointestinal	15 (41,7)	21 (58,3)	36 (100)	
Doenças hepatobiliares	12 (34,3)	23 (65,7)	35 (100)	
Hérnia inguinal	6 (30,0)	14 (70,0)	20 (100)	
Doenças cardiovasculares	8 (72,7)	3 (27,3)	11 (100)	
Doenças pulmonares	2 (33,3)	4 (66,7)	6 (100)	
Outras	1 (20,0)	4 (80,0)	5 (100)	

*χ^2^ de Pearson; p<0,05*. EMAP: espessura do músculo adutor do polegar; IMC: índice de massa corporal; ASG: Avaliação Subjetiva Global.

Foram analisadas a correlação entre EMAP e idade, PCT, CMB, AMBc, CP e IMC. Foram encontradas correlações significativas, porém fracas, entre a medida da EMAP e as variáveis antropométricas, CMB, AMBc, CP e IMC. Não foram encontradas correlações significativas entre a EMAP com a PCT e idade (Tabela 4).

**Tabela 4:** Correlação entre a espessura do músculo adutor do polegar e variáveis clássicas do estado nutricional

Variáveis (n=150)	Média	DP	IC95%		r^*^

			Inferior	Superior	
Idade	49,93	16,05	47,34	52,52	0,122
PCT	16,46	8,35	15,12	17,81	0,105
CMB	23,57	3,35	23,03	24,11	0,326†
AMBc	36,66	12,43	34,65	38,67	0,371†
CP	34,47	4,62	33,72	35,22	0,320†
IMC	25,17	5,09	24,34	25,99	0,290†

*Correlação de Pearson; †p<0,01. DP: desvio padrão; IC95%: intervalo de confiança de 95%; PCT: prega cutânea triciptal; CMB: circunferência muscular do braço; AMBc: área muscular do braço corrigida; CP: circunferência da panturilha; IMC: índice de massa corporal.

Os resultados da regressão linear múltipla são apresentados na tabela 5. Permaneceram no modelo final as variáveis gênero, CMB, AMBc e IMC, explicando 24% da medida da EMAP. A CMB foi a variável que mais influenciou nessa medida, mesmo após o ajuste para gênero, AMBc e IMC, com redução de 0,392mm.

**Tabela 5:** Regresão linear múltipla para a variável dependente espessura do músculo adutor do polegar

	EMAP		

	β	Erro padrão	Valor de p
Gênero	0,300	0,795	0,001
CMB	-0,392	0,249	0,036
AMBc	0,491	0,061	0,004
IMC	0,305	0,092	0,004

R^2^=0,238. EMAP: espessura do músculo adutor do polegar; AMBc: área muscular do braço corrigida; IMC: índice de massa corporal.

## DISCUSSÃO

Neste estudo, o estado nutricional definido a partir do IMC e da ASG apresentou correlação significativa com a EMAP. Após ajustes para as variáveis de confusão, a EMAP foi associada com gênero, CMB, AMBc e IMC. Houve diferenças significativas entre o gênero, mas não entre adultos e idosos.

Melo et al.,^7^ encontraram desnutrição em 31,8% dos pacientes do gênero masculino e em 68,2% do gênero feminino. Estes dados são corroborados por outros trabalhos da mesma natureza,^18-20^ nos quais os homens apresentaram medidas antropométricas maiores, tal qual neste estudo.

A EMAP é uma medida capaz de estimar a perda de massa muscular e possui vantagens como sua medida ser fácil, rápida e de baixo custo, além de dispensar o uso de fórmulas para calcular o compartimento muscular.^19^ Estudos^5,21-23^ têm demonstrado sua utilização em diversas condições clínicas e tratamentos, com resultados significativos quanto à sua utilização no diagnóstico nutricional, correlação com variáveis antropométricas, redução da massa magra, e associação no prognóstico de complicações hospitalares no pós-operatório e tempo de internação hospitalar.^6,24^

Os resultados aqui encontrados apresentam evidências de que a EMAP pode ser utilizada como indicador do estado nutricional e de redução da massa muscular, uma vez que se manteve associada a outras medidas antropométricas que avaliam o mesmo compartimento, como é o caso da CMB e da AMBc, além de ratificar a importância de sua utilização no pré-operatório, para que haja a identificação de risco nutricional e intervenção nutricional precoce.

A EMAP pode indicar mudanças na composição corporal e, por conseguinte, ser útil para detectar alterações precoces relacionadas com desnutrição e avaliar a recuperação nutricional.^20^

Estudo de Bragagnolo et al.^25^ avaliou 124 pacientes submetidos à cirurgia de grande porte do trato gastrointestinal e concluiu que a EMAP pode ser utilizada como preditora de complicações no pós-operatório e mortalidade, além de ser uma importante ferramenta para avaliação do estado nutricional em pacientes cirúrgicos. Este estudo ainda avaliou a associação da EMAP com a ASG, considerada um método padrão-ouro na avaliação nutricional de pacientes hospitalizados. Os resultados apresentaram correlação significativa entre elas, embora a ASG não tenha permanecido no modelo final pela análise de regressão. A CMB foi a variável que mais influenciou na medida da EMAP nesta população após os ajustes.

Estudo de da Silva et al.^26^ avaliou o estado nutricional de 43 pacientes oncológicos, maiores de 18 anos, considerando EMAP, IMC, PCT, CP, a AMBc e a força de pressão palmar dos pacientes. Houve pequena concordância entre os parâmetros utilizados, como a EMAP e ASG, mas foram obtidas diferenças significativas entre os valores de CB, AMBc, EMAP e força de pressão palmar, indicando que esses parâmetros podem ser úteis na identificação de pacientes nutridos e desnutridos, desde que sejam definidos valores de ponto de corte.

Resultados diferentes foram encontrados por Gonzalez et al.,^27^ que analisaram 361 pacientes cirúrgicos. Após análise para as variáveis de confusão, o estado nutricional avaliado pela ASG foi determinante na medida da EMAP, com importante redução de seus valores em pacientes moderadamente e gravemente desnutridos. Achados semelhantes foram encontrados por Bragagnolo et al.^5^ e Caporossi et al.^22^

Poziomyck et al.^28^ estudaram 74 pacientes adultos e idosos, submetidos à ressecção de tumores do trato gastrointestinal, com o objetivo de avaliar qual seria o método de avaliação nutricional mais sensível neste grupo. Utilizaram ASG, EMAP, IMC, CB, CMB, percentual de perda de peso e PCT, além de exames bioquímicos. Os resultados revelaram que a EMAP e a ASG são confiáveis na previsão de mortalidade e podem ser utilizadas na prática clínica.

São consensos na literatura à facilidade de aplicação, o baixo custo, a localização do músculo aferido e as diversas aplicações em que a EMAP é possível. No entanto, deve ser destacado que a ausência de um padrão de referência para as diversas condições clínicas, gênero e ciclos da vida pode gerar resultados não representativos da condição nutricional real do paciente, além da condição clínica e do estado de hidratação.

A atrofia da EMAP reflete a perda da vida laborativa. A presença da desnutrição e a doença de base podem provocar redução das atividades diárias e possível catabolismo, resultando em diminuição progressiva da EMAP.^22^

Segundo Gonzalez et al.,^20^ é importante ressaltar que pesquisas que identificam valores muito discrepantes em relação às referências indicadas nas literaturas podem estar pautadas em erro, no momento de ser pinçado o ponto anatômico correto, na calibração do plicômetro, bem como na variabilidade entre avaliadores de um mesmo estudo.

Entre as limitações deste estudo, deve ser destacado o fato de ser um estudo transversal, realizado em uma única unidade, de elevada rotatividade, com características específicas, além de não considerar as alterações bioquímicas e ser examinador dependente.

## CONCLUSÃO

Os resultados encontrados indicaram que a aferição da espessura do músculo adutor do polegar pode ser incluída na triagem nutricional de pacientes cirúrgicos, visando acelerar e facilitar o diagnóstico nutricional desses pacientes, bem como detectar a depleção proteica. A espessura do músculo adutor do polegar tem se mostrado uma medida confiável e que pode ser implementada no processo da avaliação nutricional, pois é capaz de identificar o risco de desnutrição, juntamente de outras variáveis e métodos de avaliação nutricional. Todavia, são necessárias novas pesquisas, para identificar as razões de diferentes achados na literatura, principalmente no que tange ao ponto de corte para avaliação de pacientes cirúrgicos, considerando faixa etária e sexo.
